# Role of Extracellular Vesicles in Cell Death and Inflammation

**DOI:** 10.3390/cells10102663

**Published:** 2021-10-05

**Authors:** Rahul Sanwlani, Lahiru Gangoda

**Affiliations:** 1 Department of Biochemistry and Genetics, La Trobe Institute for Molecular Science, La Trobe University, Melbourne, VIC 3083, Australia; 19168073@students.latrobe.edu.au; 2 The Walter and Eliza Hall Institute of Medical Research (WEHI), 1G Royal Parade, Parkville, Melbourne, VIC 3052, Australia; 3 Department of Medical Biology, University of Melbourne, Parkville, Melbourne, VIC 3010, Australia

**Keywords:** extracellular vesicles, exosomes, microvesicles, apoptotic bodies, apoptotic extracellular vesicles, cell death, inflammation, sepsis, lung inflammatory disorders, SARS-CoV-2

## Abstract

Extracellular vesicles (EVs) have been identified as novel mediators of intercellular communication. They work via delivering the sequestered cargo to cells in the close vicinity, as well as distant sites in the body, regulating pathophysiological processes. Cell death and inflammation are biologically crucial processes in both normal physiology and pathology. These processes are indistinguishably linked with their effectors modulating the other process. For instance, during an unresolvable infection, the upregulation of specific immune mediators leads to inflammation causing cell death and tissue damage. EVs have gained considerable interest as mediators of both cell death and inflammation during conditions, such as sepsis. This review summarizes the types of extracellular vesicles known to date and their roles in mediating immune responses leading to cell death and inflammation with specific focus on sepsis and lung inflammation.

## 1. Extracellular Vesicles: Introduction, Subtypes, and Cargo

Extracellular vesicles (EVs) have been defined as nanosized vesicles that are shed into extracellular space [1]. First identified in differentiating reticulocytes three decades ago, EVs were thought to be the waste disposal system of cells which aided in the elimination of cellular waste [2,3]. However, extensive studies over the past two decades have been crucial for introducing a paradigm shift by ascertaining a more sophisticated role for EVs [4]. Intercellular communication is no more thought to be a consequence of only direct cell-cell contact or secreted factors such as hormone signaling. EVs are now known to mediate multiple pathophysiological processes by facilitating intercellular communication [5]. Their expansive role in health and disease has been investigated in multiple models [6,7]. As more studies continue to shed light on the diverse role played by EVs, their role is now becoming apparent to be crucial in not just mediating communication between distant cells in an individual but also in facilitating the transfer of bioactive compounds inter-individual, cross-species and even inter-kingdom [8,9,10]. 

**Figure 1:**
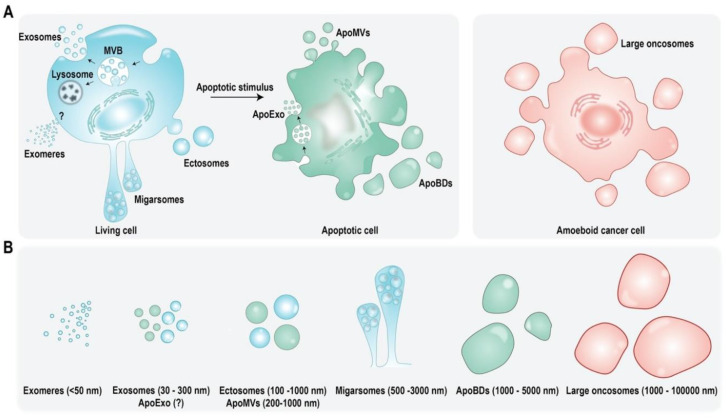
Schematic representation of EV subtypes identified in eukaryotic cells. (**A**) Although live cells were initially thought to shed exosomes and microvesicles, recent developments have identified novel EV subtypes including exomeres and migrasomes, whereas cancer cells also partake in secretion of large oncosomes. On the contrary, apoptotic cells which were long though to shed only ApoBDs are now known to secrete other EV subtypes including ApoMVs and ApoExos. (**B**) Various EV subtypes shed by live or apoptotic cells are distinct in their biogenesis, cargo, origin, and size.

Multiple subtypes of eukaryotic EVs exist and they are primarily categorized based on their biogenesis, subcellular origin, and size (Figure 1) [1,11]. Exosomes are vesicles that are endocytic in origin and shed as a consequence of multivesicular bodies (MVB) or late endosomes fusing with the plasma membrane [12]. They range in size from 30 to 150 nm and were believed to be the smallest EV subtype until the recently [1,13]. Exomeres have been identified as secretory nanoparticles that are less than 50 nm in size with their mode of biogenesis currently unknown [14,15], whereas shedding microvesicles (MVs) are larger in size and may range from 100 to 1000 nm. They originate due to budding or outward vesiculation of plasma membrane, thus also referred to as ectosomes [1,16]. Furthermore, there are new classes of EVs defined, such as migrasomes and large oncosomes [17,18,19]. Up until now, apoptotic cells were thought to only shed apoptotic bodies (ApoBDs) which are similar to microvesicles in origin as they emerge due to budding of plasma membrane. However, ApoBDs are larger in size and may range from 50 to 5000 nm [20,21]. More recently, apoptotic cells were found to partake in secretion of other EV subtypes and apoptotic cell-derived EVs (ApoEVs) now include apoptotic microvesicles (ApoMVs) and apoptotic exosomes (ApoExos) which are physiologically distinct from ApoBDs [22]. Similar to MVs, ApoMVs range in size from 200 to 1000 nm and also called apoptotic microparticles. They have a higher membrane integrity than ApoBDs, promoting better molecular exchange [22,23,24], whereas ApoExos are the most recent addition to ApoEVs and were initially reported to be released by endothelial cells following caspase 3-dependent MVB formation [25,26]. Though there is limited knowledge regarding their origin and function, they continue to be explored in-depth as final messengers of apoptotic cells [22]. 

EVs contain a diverse cargo comprising of a plethora of proteins, nucleic acids and lipids [27]. EV cargo is capable of mediating a variety of responses in the recipient cell including infections [28], signal transduction [29] and immune regulation [30]. EV cargo is not only different for various subtypes of EVs but also varies between same EV subtype derived from two different cell types [31,32]. EV proteome is its most well studied cargo so far [27]. Exosomes have been particularly found to be enriched with endosome associated proteins with documented roles in vesicle trafficking, such as Rabs, Annexins, and Flotillins. Furthermore, Alix and TSG101 have been previously claimed as molecular markers of exosomes [5,12]. However, even though these proteins with a role in MVB biogenesis are enriched in exosomes, they are not markers as previously claimed [1]. Exosomes have also been found to be enriched with heat shock proteins (HSPs), and membrane proteins, such as integrins, major histocompatibility complex (MHC) class I and II and tetraspanins CD63, CD9, and CD81 [12,32,33]. MVs on the other hand, having reported roles in metastases, invasion, and inflammation, are enriched with proteins, such as Annexins, selectins, CD40, and metalloproteinases [34,35]. Interestingly, even though ApoEVs have been suggested to have characteristics similar to EVs released by healthy cells, their cargo may enable more distinctive roles. With their unique cargo, ApoEVs may influence processes such as inflammation and autoimmunity in addition to aiding in clearance of apoptotic by-products and remnants. Furthermore, ApoBDs not only contain biomolecule cargo from the originating cell but can also contain nuclear fragments and cellular organelles, such as mitochondria and endoplasmic reticulum [20,22,36,37]. Identification of nucleic acids including mRNA, miRNA (miR), and DNA in EVs has generated significant interest [38,39]. Functional transfer of nucleic acids via EVs is known to regulate recipient cells and control signaling pathways [40]. Lastly, EVs are abundant in lipids and lipid-related enzymes including but not limited to sphingomyelin, ganglioside, ceramide, cholesterol, and prostaglandins. In fact, EV lipid composition has been observed to be different than that of the cell type of origin [41,42,43]. Similar to proteins and nucleic acids, lipids in EVs also have functional role in controlling signaling pathways in recipient cells and thus contribute in more ways than only providing structural integrity to these vesicles [44]. 

## 2. EVs as Regulators of Cell Death

**Figure 2:**
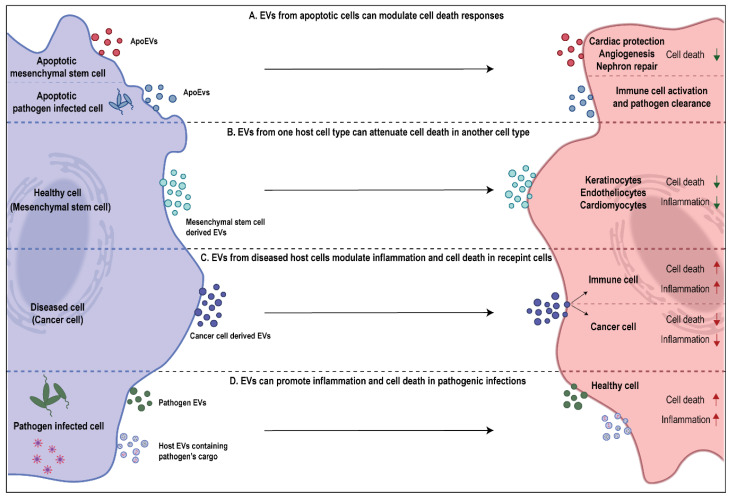
Context dependent roles of EVs in mediating cell death and inflammation. (**A**) ApoEVs have the ability to either attenuate or promote cell death. MSC derived ApoEVs have regenerative function whereas ApoEVs from pathogen infected cells induce activation of immune cells, leading to enhanced targeting and clearance of pathogen cells. (**B**) Similarly, EVs from healthy MSCs have regenerative function in preventing cell death and inflammation in various cell types. (**C**) Contrastingly, EVs from diseased host cells may function in different ways depending on the recipient cell. For instance, cancer cell derived EVs promote cell death and inflammation in immune cells but prevent cell death and inflammation in other cancer cells. (**D**) Infected cells can shed pathogen derived EVs or host EVs loaded with pathogen’s cargo. These EVs can promote cell death and inflammation in recipient host cells.

Cell death is an important process that involves the removal of cells during certain developmental stages or those that are compromised due to exposure to toxins. Cell death can also occur as a result of natural processes, where old cells die and are replaced by new ones to maintain homeostasis in an individual. On the contrary, it could also be a result of unnatural factors, such as pathological conditions or local injuries [45]. As established above, cells undergoing apoptosis can shed multiple EV subtypes aiding in not only getting rid of apoptotic debris but also facilitating communication with other cell types. However, the role of EVs in cell death is not only limited to the functions of EVs shed by apoptotic cells. As more studies continue to understand the regulatory potential of EVs, it is becoming increasingly evident that EVs from living cells are also capable of moderating cell death responses in recipient cells (Figure 2). The cargo embodied in EVs is not just dependent on the EV subtype but also the cell type of origin, such as diseased versus healthy cells [13,46]. Thus, in a context dependent manner, these signaling moieties may be capable of promoting or attenuating cell death in a particular recipient cell type. The subsequent sections are aimed at elaborating the role of EVs in cell death in various pathophysiological conditions (Tables 1 and 2). As this review specifically addresses the role of EVs in inflammatory disorders, this section would emphasize the role of EVs in mediating immune and inflammatory cell death.

## 3. Role of EVs in Attenuating Cell Death

Attenuation of cell death via extracellular vesicles has been studied in multiple models (Table 1). It has recently been discovered that milk-derived EVs (MEVs) have a role in promoting gut health and development upon uptake by intestinal epithelial cells (IECs) [9]. MEVs from multiple sources, such as porcine, bovine and humans have been demonstrated to prevent cell death in IECs during oxidative stress and hypoxia and promote their survival and proliferation [47,48,49]. This ability of MEVs has been attributed to its cargo inhibiting p53 expression in IECs [50]. However, the role of MEVs in attenuation of cell death may not always be beneficial for the consumer. miR-148a and miR-21 which are a part of MEV cargo have been designated as oncomiRs due to their role in promoting vital cellular processes, such as proliferation and survival by inhibition of tumor suppressor genes [51,52,53]. Thus, the ability of MEVs to attenuate cell death could be detrimental too. Multiple studies have now demonstrated the ability of tumor cell-derived EVs in promoting survival and preventing cell death in recipient tumor cells (Figure 2). For instance, exosomes from N-myc amplified neuroblastoma cells were found to enhance survival by inducing chemoresistance in non-N-myc amplified cells, preventing doxorubicin induced apoptosis [54]. Furthermore, in other studies using glioblastoma models, it has been shown that exosomes shed by apoptotic tumor cells were able to confer survival advantage to the surviving glioblastoma cells by transferring spliceosome components [55]. Another model where EVs attenuated cell death, that continues to be increasingly defined with studies, is that of stem cells. The therapeutic nature of mesenchymal stem cells (MSCs) due to paracrine signaling is well established [56]. Recently, it was observed that the MSC derived therapeutic benefits were mediated largely due to EVs. MSC-derived EVs were reported to enhance proliferation and migration of keratinocytes and suppress apoptosis, thus promoting enhanced healing at wound sites [57]. Similarly, MSC-derived EVs are now well known for their cardioprotective function. They have been found to inhibit apoptosis, autophagy and fibrosis by modulating cardiomyocytes and endotheliocytes. Further, MSCs were also found to stimulate cell proliferation and angiogenesis, thus leading to enhanced cardiac survival (Figure 2) [58,59,60].

Attenuation of cell death is not necessarily a feature of EVs shed by live cells. ApoEVs which serve as the last messages from dying cells have been thoroughly characterized for the complex role they play in tissue development, regeneration and apoptosis (Figure 2) [65]. Similar to MSC-derived exosomes, MSC-derived ApoEVs have been shown to promote angiogenesis and the recovery of cardiac function via the regulation of autophagy [61]. Further, apoptotic MSC-derived EVs have also been found to have a role in nephron repair following injury. These MSC-derived ApoEV associated benefits could be attributed to compensatory proliferation signaling which has been implied to be consequence of apoptosis [62]. Lastly, ApoEVs released by host cells have been found to indirectly attenuate cell death in surrounding cells by aiding in the regulation of immune system and preventing the spread of pathogen in diseases settings. For instance, ApoMVs shed by macrophages infected with *Mycobacterium tuberculosis* upon being engulfed by dendritic cells induced antimicrobial immunity. As a consequence, it led to enhanced clearance of the pathogen and prevented further infection of macrophages [63]. A similar phenomenon has also been discovered in the clearance of prion infection by microglia cells upon engulfment of ApoBDs from prion infected neurons [64]. Overall, the ability to EVs to prevent cell death directly or indirectly due to its signaling cargo is evident. These findings highlight the broad untapped therapeutic potential of EVs in controlling diseases and in treating pathological conditions that have so far been extremely challenging to tackle. 

## 4. Role of EVs in Promoting Cell Death

The role of EVs in pathological diseases has been extensively studied in host-pathogen interaction model. EVs have evolved as means of communication between host and pathogen by mediating inter-kingdom transfer of cargo. An increasing number of studies over the last decade have highlighted the ability of EVs in facilitating the establishment and accelerated spread of infections in the host. The pathogens have been found to either work by hijacking and exploiting host’s machinery or even deploying their own EVs to suppress the host’s immune system (Figure 2) [66,67,68,69]. For instance, astrocytes infected with Human immunodeficiency virus (HIV) have been shown to shed EVs with the viral Tat protein, leading to neuronal cell death [70]. Similarly, Epstein–Barr virus has been shown to deploy viral components, such as latent membrane protein-1 in EVs shed by infected carcinoma cells, ultimately leading to the alteration of signaling pathways and cell death in T-lymphocytes [71]. Bacterial pathogen *Mycobacterium tuberculosis* too has been shown to hijack host cell’s EV machinery and load virulent cargo in EVs shed by infected macrophages. These EVs were capable of inducing apoptosis in Jurkat T cells [72]. Macrophages infected with bacterial pathogens including *Mycobacterium tuberculosis, Salmonella typhimurium,* and *Toxoplasma gondii* have been shown to shed small EVs which contain pathogen associated molecular patters (PAMPs). Upon uptake by uninfected macrophages, these EVs were responsible for elevated cytokine release and resulting pro-inflammatory response [66]. Additionally, it has been shown that EVs shed by a variety of bacterial pathogens themselves harbor virulent cytotoxic cargo which enables them to establish infection in healthy cells and suppress host’s defenses [67]. For instance, Gram negative bacterial EVs have been shown to aid in the delivery of lipopolysaccharide (LPS) to the recipient cell’s cytosol. This event induces an inflammatory form of cell death, known as pyroptosis, by triggering casapase-11 dependent effector responses [73]. Furthermore, *Neisseria gonorrhoeae* derived EVs have been shown to contain the Porin protein PorB, which induces apoptosis in host macrophages [74]. Similarly, the presence of Shiga toxin 2a has been observed in pathogen derived EVs and found to have cytotoxic implications via caspase-9 and caspase-3 activation in human IECs [75]. 

The role of EVs as promoters of cell death has been elucidated in other pathological conditions too, such as cancer, autoimmunity, and pulmonary disorders (Figure 2). For instance, in a recent study, the role of T-lymphocyte derived exosomes in Type-1 diabetes was explored. Specific miR (miR-142-3p, miR-142-5p, and miR-155) have been found to be enriched in T-lymphocyte derived EVs which led to the elevated incidence of Type-1 diabetes as they selectively targeted and induced apoptosis in pancreatic β cells along with chemokine signaling including *Ccl2, Ccl7,* and *Cxcl10* [76]. Tumor cell derived EVs have also been found to use this mechanism to suppress immune response. A recent study showed that exosomal PD-L1 was upregulated and carried in EVs released by metastatic melanomas. These EVs were found to have role in exhaustion of CD8^+^ T cells which aided the tumor cells in evading immune response [30]. Similarly, sera EVs derived from individuals with oral squamous cell carcinoma had elevated expression of Fas ligand which led to T cell apoptosis [77]. In context of lung disorders, such as asthma, airway inflammatory potential of eosinophil derived EVs due to pro-inflammatory cargo has been observed [78]. Further, these EVs were demonstrated to lead to apoptosis in primary alveolar epithelial cells by interfering with JAK/STAT signaling [79]. Similarly, monocyte derived EVs encapsulated caspase-1 and their ability to induce apoptosis of pulmonary microvascular endothelial cells was observed in acute lung injury (ALI) and acute respiratory distress syndrome (ARDS) [80,81]. Another intriguing observation of EV mediated lung pathology and cell death was made in chronic obstructive pulmonary disease (COPD). The apoptotic endothelial cells during COPD shed EVs loaded with inflammatory cargo that promote progression of the disease by inducing apoptosis in otherwise healthy endothelial cells [82]. The subsequent sections further discuss the role of EVs in promoting lung inflammatory disorders in more detail.

The immense potential of EVs as mediators that promote cell death in various pathological conditions has been concretely established (Table 2) and continues to be explored in depth. Collectively, these studies present opportunities to exploit EVs at a therapeutic front for multiple purposes. These findings are key in devising novel therapeutic paradigms and alternatives in treating and preventing the spread of infections, as well as diseases, such as cancer, by targeting EV mediated signaling. Although promising, these studies need further validation with better in vivo models and improved understanding of the molecular mechanisms and cargo components of interest before extrapolating the use of EVs to clinical setups. 

## 5. EVs in Inflammation

Inflammation is the immune system’s response to harmful stimuli, such as pathogens, damaged cells, toxic compounds, or irradiation [83]. Some forms of cell death can provoke inflammatory responses. Although apoptosis is a immunologically silent process and generally does not trigger inflammation, other forms of lytic cell death, such as necrosis and pyroptosis, are pro-inflammatory [84]. For instance, cells infected with pathogenic bacteria or viruses undergo pyroptosis induced by activation of inflammasome sensors, resulting in the loss of plasma membrane integrity, leakage of cellular contents, and inflammation [85]. Inflammation can be further classified as either acute inflammation which occurs immediately after injury and lasts for few days or chronic inflammation which may last for several months or sometimes even years. During acute inflammatory responses, the body efficiently minimizes impending damage by removing injurious stimuli and initiating the healing process. This leads to the restoration of tissue homeostasis and the resolution of inflammation [86]. Another form of cell death known as pyroptosis has been long studied for its role in chronic inflammatory diseases, such as Alzheimer’s disease [87,88] and atherosclerosis [89]. Under chronic conditions, the inflammation fails to resolve due to excessive and uncontrollable activity of the immune system which can lead to extensive tissue damage [90]. Hence, new therapies aimed at preventing this hyperactivity of the immune system could have major clinical benefits. 

Recently EVs have gained attention as mediators and biomarkers of inflammation (Table 3). The discovery of MHC harboring EVs shed by multiple lineages of immune cells suggested a complex functional role for these vesicles as they mediated antigen specific immune response [34,91,92]. Further, secretion of tumor growth factor β1 via EVs was found to modulate anti-inflammatory effects [93]. ApoExos containing Sphingosine 1-Phosphate Receptors 1 and SPR1/3 have been identified to induce proinflammatory cytokine production in macrophages leading to activation of NF-κB and p38 MAPK [37]. Non-coding RNAs delivered via ApoExos have been found to be pro-inflammatory in nature as they stimulate toll-like receptors [94]. Additionally, EVs have been found to contain wide variety of proteins, including chemokines and inflammatory cytokines, such as tumor necrosis factor (TNF), IL-1β, CXCL2, and CXCL8 suggesting an important immune modulatory functions of EVs [95,96,97]. In the following section, we aim to discuss existing literature with the focus on the role of EVs during acute inflammation in diseases, such as sepsis and ARDS, and chronic inflammation associated with diseases, such as COPD and asthma.

## 6. EVs in Sepsis Associated Inflammation

Sepsis is a dysregulated systemic inflammatory disorder that can lead to life threatening organ injury [124]. Since their discovery over two decades ago, Evs originating from different type of cells have been shown to play diverse roles in sepsis. The source of Evs during sepsis can be either from the pathogen responsible for the infection or the host’s own cells [125]. 

Cell death is the most common outcome during infections. Pathogen can trigger both non-inflammatory modes of cell death, such as apoptosis and autophagy or inflammatory modes of cell death, such as pyroptosis and oncosis [126]. Gram-positive and Gram-negative bacteria, as the most frequent infectious agent of sepsis, can produce EVs [127] that carry bacterial virulence factors that contribute towards the systemic inflammation [98]. During sepsis, lymphocytes are depleted by apoptosis, which leads to immunosuppression [128]. Exosomes from HIV type 1 infected cells has been shown to contain the accessory extracellular viral protein Nef, which was shown to induce apoptosis of CD4^+^ T-cells [99]. Given that pathogen-derived EVs carry cargo that are representative of their cell of origin, bacterial protoplast-derived nanovesicles have been used to vaccinate mice against these pathogens in models of pneumonia and peritonitis [129].

On the other hand, host-derived EVs during sepsis are produced mainly from immune cells such as platelets and innate immune cells [125]. Increased levels of platelet MVs has been observed in patients with Gram-negative bacterial sepsis [130]. MVs originating from platelets or granulocytes were also elevated in meningococcal sepsis [100]. These MVs were associated with increased coagulation activity. Consequently, MVs have also been linked with thromboembolic events and in patients with transient ischemic attacks, lacunar infarcts and multi-infarct dementias have elevated levels of MVs [131,132,133]. Hence, host-derived MVs have been suggested as a novel target for therapeutic intervention in clinical conditions with enhanced coagulation activation [100]. In sepsis neutrophils are one of the vital leukocytes to play first line of defense [134,135,136]. Neutrophil-derived EVs dramatically increased in the blood of mice subjected to cecal ligation and puncture (CLP), a commonly used animal model of sepsis [137]. Treatment with neutral sphingomyelinase inhibitor, GW4869 which blocks exosome production, significantly improved the survival of mice subjected to LPS injection or CLP, suggesting that exosomes might play an important role in sepsis [138]. Studies have also shown altered contents and function of host derived EVs during sepsis. In sepsis, exosomes carry increased levels of cytokines and damage-associated molecular patters (DAMPS) to induce inflammation. It is well documented that patients with sepsis have increased levels of serum cytokines which are important for initiation of the “cytokine storm”. Exosomes have been shown to carry biologically active pro-inflammatory cytokines IL-12, IL-15, IL-17, and IFN-γ at early phase and anti-inflammatory cytokines, IL-4 and IL-10 at late phase of the cytokine storm [101,102].

EVs play major roles in organ damage during sepsis. Activated polymorphonuclear leukocytes from septic patients have been shown to produce microparticles with increased adhesion molecules that can activate the vascular endothelium leading to endothelial injury and resultant organ dysfunction [103,104]. Moreover, platelet-derived exosomes from sepsis patients induced apoptosis of endothelial cells and vascular smooth muscle cells due to increased NADPH activity and by generating reactive oxygen species, such as superoxide, nitric oxide, and peroxynitrite [105]. Thereby, exosomes have been implicated in the induction of oxidative stress and myocardial dysfunction and vascular cell apoptosis. Additionally, exosomes have also been shown to contribute to septic encephalopathy directly and by causing systemic immune dysfunction due to the collapse of neuroendocrine immune networks [139]. Lastly, exosomes may contribute to chronic liver dysfunction after sepsis, as exosomal miR-103-3p from LPS-activated macrophages targeted KLF4 to increase liver fibrosis [106].

The role of EVs in sepsis may not be limited to causing damage to host cells and organs. Interestingly, several protective host EV-related mechanisms were recently discovered in sepsis models. In several studies, granulocyte derived EVs have shown antibacterial effects [107] in addition to supporting the production of inflammatory mediators and protecting from vascular dysfunction [108]. Likewise, EVs containing alpha-2-macroglobulin excreted by neutrophilic granulocytes were shown to help in bacterial clearance and reduce inflammation in a mouse model of sepsis [109]. Immature dendritic cell-derived exosomes attenuated the acute systemic inflammatory response in sepsis by enhancing apoptotic cell clearance in septic rats [110]. Overall, EVs have both protective and detrimental roles during sepsis in a contextual manner, depending on their cell type of origin and the cargo contained within [125]. In this context, the protein cargo from EVs isolated from the serum of patients following sepsis diagnosis has been analyzed in a clinical study to identify biomarkers for better disease management and monitoring disease progress and prognosis [140]. Further knowledge from similar studies will be instrumental in developing novel therapeutic interventions and even biomarker discovery for improved management of sepsis patients.

Lung is one of the most susceptible organs to systemic inflammation. Septic patients are often accompanied by ALI or ARDS, which further add to sepsis-associated mortality [141]. Thus, in the following section we discuss the role of EVs in lung inflammation.

## 7. EVs in Lung Inflammatory Disorders

Lung is the organ with highest vascular density in the human body. Hence, the endothelium of the lung contributes substantially to the circulation of EVs which, in turn, play critical roles in normal lung physiology [142]. Lung epithelial cell-derived exosomes contain membrane-tethered mucins that contribute to the innate defense of the airway [111]. EVs have also gained attention as novel communicators in lung diseases, such as COPD, asthma, ALI, and ARDS [143]. Alveolar macrophages can secrete SOCS1 and 3 (suppressor of cytokine signaling 1, 3) in EVs which can further control inflammatory signaling. This process has been observed to be dysregulated in association with cigarette smoke exposure [112]. EV secretion from lung endothelial cells is enhanced during adverse conditions, such as infection and smoke exposure [144,145]. These vesicles are primarily generated by an apoptosis-dependent mechanism that is triggered upon pulmonary endothelial damage. Circulating lung endothelial cell-derived EVs can, therefore, be potential biomarkers for lung injury and inflammation. In one such study, cigarette smoke extract induced CCN1-enriched exosomes. CCN1 is generally known to play crucial roles in tissue remodeling and repair as an early response gene product and as an extracellular matrix protein [146,147]. However, these exosomes were found to induce the activation of IL-8 secretion which recruits inflammatory cells, particularly neutrophils, into the lung parenchyma, further promoting lung inflammation in COPD [113]. 

Enhanced secretion of exosomes by lung epithelial cells has also been observed in asthma which is a chronic, recurrent, and incurable allergy-related respiratory disease. These epithelial cell-derived exosomes induced proliferation and chemotaxis of undifferentiated macrophages and inhibition of exosome production, which resulted in a reduced population of proliferating monocytes and alleviation of various asthmatic features [114]. Asthma is characterized by the infiltration of airway with T lymphocytes, mast cells, basophils, macrophages, and eosinophils [148]. Mast cells secrete exosomes that carry immunologically relevant molecules, such as MHC class II, CD86, LFA-1, and ICAM-1. These exosomes can act as messengers to induce splenic B and T cell blast activation and have been suggested to play a role in the recruitment of these cells to the lungs, eventually contributing to lung inflammation [115]. On the contrary, mast cell exosomes containing high-affinity IgE receptors (FcεRI) have been shown to suppress allergic reactions by binding to free IgE [116]. Eosinophils are the main effector cells in asthma. Eosinophils of asthmatic patients were shown to produce more exosomes than those of healthy individuals and these exosomes promoted inflammatory behavior of eosinophils that is related to asthma pathogenesis [79,117]. Similarly, dendritic cell and B cell–derived exosomes have been shown to play a role in stimulating the allergic immune response via stimulation of T cell proliferation and induction of cytokine production of allergen-specific T cells (IL-4, IL-5, and IL-13 and lower levels of IFN-γ and TNF-α) [118,119].

ALI and ARDS remain as life-threatening diseases in critically ill patients. ALI and ARDS can be triggered by both non-infectious (sterile) and infectious stimuli underlying clinical disorders, such as sepsis, pneumonia, trauma, and pancreatitis [149,150,151]. Studies have shown that proceeding sterile stimuli, alveolar type-I epithelial cells were the main source of the EVs in the bronchoalveolar lavage fluid (BALF) whereas infectious stimuli-induced BALF EVs were mainly derived from alveolar macrophages. Both sterile stimuli-induced EVs and infection-induced EVs facilitated classic macrophage activation, and subsequently promoted inflammatory lung responses via different signaling pathways [152]. Caspase-1 encapsulated in exosomes released from LPS-challenged macrophages induced lung endothelial cell apoptosis, indicating their contribution to the disruption of the alveolar-capillary barrier [81]. Given the ability of EVs to induce an inflammatory response and cell death of lung tissue, they have the potential to be targeted for more effective therapeutic approaches to treat ALI and ARDS [153].

The novel coronavirus disease (COVID-19) is responsible for millions of deaths worldwide with the respiratory failure been the most common clinical presentation [154]. Recent evidence points to a correlation between COVID-19 severity and pyroptotic inflammasome activation by SARS-CoV-2 infection [155]. COVID-19 can cause lung complications, such as pneumonia, and in the most severe cases ARDS and sepsis [156]. In a recent proteomic analysis of COVID-19 patient-derived exosomes, several molecules were identified which are involved in several biological processes. These processes include immune response, inflammation, and activation of the coagulation and complement pathways, the main mechanisms of COVID-19–associated tissue damage and multiple organ dysfunctions. Several potential biomarkers, such as fibrinogen, fibronectin, complement C1r subcomponent, and serum amyloid P-component that are well correlated with the COVID-19 disease’s severity were also encapsulated within these exosomes [157]. The presence of viral material amongst the exosomal cargo showed that SARS-CoV-2 could use the cell-to-cell communication system to spread infection in the host [120,157]. Cells that express ACE2 and CD9 can transfer these viral receptors to other cells via EVs, making recipient cells more susceptible for SARS-CoV-2 infection [120]. GM3-enriched exosomes were shown to be positively correlated with COVID-19 disease severity, suggesting they may partake in pathological processes related to COVID-19 pathogenesis [158]. In SARS-CoV-2 infection, the systemic inflammatory response was shown to result in the release of substantial amounts of procoagulant EVs that may act as clotting initiation agents, contributing to disease severity [121,122]. On the other hand, exosome-based strategies were also proposed to treat COVID-19 or prevent SARS-CoV-2 infection [120,123,159]. Especially, MSCs-derived EVs from a broad range of sources, including bone marrow, adipose tissue, umbilical cord are currently under investigation for many conditions and areas of regenerative medicine [160]. They have been shown to alleviate severe inflammation (cytokine storm) and repair damaged lung cells in COVID-19 by delivery of anti-inflammatory molecules. There are several ongoing clinical trials that apply these therapeutically beneficial EVs for treatment of COVID-19 patients as drug delivery platforms or vaccines [154,161,162]. Therefore, similarly to sepsis, EVs from diverse sources exhibit distinct effects during COVID-19 infections [163].

## 8. Conclusions

Overall, there is strong evidence to support the significance of EVs in the modulation of inflammation and cell death and the diseases related to these processes. EVs have been shown to play critical roles in both promoting and suppressing cell death and inflammation leading to beneficial, as well as detrimental, health outcomes. Over the last couple of decades, studies on EVs have shown their great potential to improve diagnosis and therapy in human diseases. As has been occasionally discussed in this article as well, EVs present as a valuable target for disease treatment, targeted drug-delivery, vaccine development and biomarker discovery. Furthermore, there is a need for developing better methods for isolation of pure EV subtypes and address the challenge of EV heterogeneity in preparations. The establishment of precise isolation methods is crucial before a particular function can be attributed to any specific EV subtype. Further, MSC-derived EVs are widely explored in preclinical and clinical settings due to their therapeutic potential. However, translation of such findings regarding EVs to clinical practices would require further exploration of their exact mode of biogenesis and cargo components, which are responsible for the desired phenotype to clearly understand the underlying mode of action. Despite these knowledge gaps and shortcomings, with further exploration employing improved methodologies and techniques, EVs remain as putative therapeutic targets for the future.

## Figures and Tables

**Table 1:** Table summarizing the studies which elucidate the role of EVs in attenuation of cell death. NS—not specified in the original article.

Cargo	EV Source	Function	Reference
NS	Milk (bovine, porcine, human)	Attenuate cell death in IECs	[47,48,49,50]
miR-148a, miR-21	Milk (bovine)	OncomiRs, inhibition of tumor suppressor genes	[51,52,53]
NS	N-myc amplified neuroblastoma cells	Chemoresistance and enhanced survival in non N-myc amplified tumor cells	[54]
Spliceosome components	Apoptotic glioblastoma cells	Enhanced survival in recipient tumor cells	[55]
NS	MSCs (exosomes)	Enhance proliferation and survival of keratinocytes, cardiomyocytes and endotheliocytes	[57,58,59,60]
NS	MSCs (ApoEVs)	Regulation of cardiac function and autophagy, promote nephron repair	[61,62]
NS	Pathogen infected host cells	Immune regulation and enhanced pathogen clearance to prevent spread of infection	[63,64]

**Table 2:** Table summarizing the studies which elucidate the role of EVs in promoting cell death. NS—not specified in the original article.

Cargo	EV Source	Function	Reference
HIV Tat protein	HIV infected astrocytes	Neuronal cell death	[70]
Latent membrane protein-1, galectin-9	Epsetien-Barr virus infected nasopharyngeal carcinoma cells	T lymphocyte cell death	[71]
NS	*Mycobacterium tuberculosis* infected T-lymphocytes	Apoptosis in Jurkat T cells	[72]
PAMPs	Pathogen infected macrophages	Pro-inflammatory cytokine release in uninfected macrophages	[66]
LPS	Gram negative bacteria	Caspase-11 mediated pyroptosis in	[73]
PorB	*Neisseria gonorrhoeae*	Apoptosis in macrophages	[74]
Shiga toxin 2a	Escherichia coli O104:H4	Cell death in IECs	[75]
miR-142-3p, miR-142-5p and miR-155	T-lymphocytes	Selective targeting and apoptosis of pancreatic β cells leading to type-1 diabetes	[76]
PD-L1	Metastatic melanomas	Exhaustion of CD8^+^ T cells leading to immune response evasion	[30]
Fas ligand	Serum from patients with oral squamous cell carcinoma	CD8^+^ T cell apoptosis	[77]
NS	Eosinophils	Airway inflammation and apoptosis in primary alveolar epithelial cells	[78]
Caspase-1	Monocytes	Apoptosis of pulmonary microvascular endothelial cells in ALI and ARDS	[80,81]
NS	Apoptotic endothelial cells	Apoptosis in healthy endothelial cells in COPD	[82]

**Table 3:** Table summarizing studies with a role for EVs in mediating inflammation sepsis and lung disorders. NS—not specified in the original article.

Cargo	EV Source	Identified in	Function	Reference
MHC	Dendritic cells, B lymphocytes	NS	antigen specific immune response	[34,91,92]
NS	Neutrophils	NS	anti-inflammatory effects	[93]
Sphingosine 1-Phosphate Receptors 1 and SPR1/3	Bone marrow-derived macrophages	NS	pro-inflammatory effects	[37]
Non-coding RNA	Endothelial cells	NS	pro-inflammatory effects	[94]
Chemokines and inflammatory cytokines: TNF, IL-1β, CXCL2, CXCL8	Dendritic cells, Mesenchymal stromal cells	NS	immune modulatory functions	[95,96,97]
Bacterial virulence factors	Bacteria	Sepsis	pro-inflammatory effects	[98]
Nef protein	HIV type 1 infected cells	HIV type 1 infection	anti-inflammatory effects	[99]
NS	platelets, granulocytes	Meningococcal sepsis	pro-coagulation activity	[100]
IL-12, IL-15, IL-17, IFN-γ	NS	sepsis	pro-inflammatory effects	[101,102]
IL-4, IL-10	NS	sepsis	anti-inflammatory effects	[101,102]
Adhesion molecules	activated polymorphonuclear leukocytes	sepsis	organ damage	[103,104]
NS	platelet	sepsis	pro-apoptotic	[105]
miR-103-3p	activated macrophages	sepsis	organ damage	[106]
NS	granulocyte	sepsis	anti-bacterial effects	[107]
NS		sepsis	protecting from vascular dysfunction	[108]
alpha-2-macroglobulin	granulocytes	sepsis	bacterial clearance, anti-inflammatory effects	[109]
NS	Immature dendritic cells	sepsis	anti-inflammatory effects	[110]
membrane tethered mucins	Lung epithelial cells	-	innate defense	[111]
SOCS1 and 3	Alveolar macrophages	cigarette smoke exposure	modulation of inflammatory signalling	[112]
CCN1	lung epithelial cells	COPD	pro-inflammatory effects	[113]
NS	lung epithelial cells	Asthma	pro-inflammatory effects	[114]
MHC class II, CD86, LFA-1 and ICAM-1	mast cells	Asthma	pro-inflammatory effects	[115]
FcεRI	mast cells	Asthma	anti-inflammatory effects	[116]
NS	eosinophils	Asthma	pro-inflammatory effects	[79,117]
NS	Dendritic cells	NS	pro-inflammatory effects	[118]
NS	B cell	Allergy	pro-inflammatory effects	[119]
Caspase-1	activated macrophages	NS	pro-apoptotic	[81]
ACE2, CD9	SARS-CoV-2 infected cells	COVID-19	promote infection	[120]
NS	NS	COVID-19	pro-coagulation activity	[121,122]
NS	mesenchymal stem cells	NS	anti-inflammatory	[123]

## Abbreviations

ALI	Acute lung injury
ApoBDs	Apoptotic bodies
ApoEVs	Apoptotic EVs
ApoExos	Apoptotic exosomes
ApoMVs	Apoptotic microvesicles
ARDS	Acute respiratory distress syndrome
BALF	Bronchoalveolar lavage fluid
CD	Cluster of differentiation
CLP	Cecal ligation and puncture
COPD	Chronic obstructive pulmonary disorder
EVs	Extracellular vesicles
HSP HIV	Heat shock protein Human immunodeficiency virus
IECs	Intestinal epithelial cells
LPS	Lipopolysaccharide
MEVs	Milk-derived EVs
MHC	Major histocompatibility complex
miR	micro-RNA
MSCs	Mesenchymal stem cells
MVB	Multivesicular bodies
MVs	Microvesicles
PAMPs	Pathogen associated molecular patters
TNF	Tumor necrosis factor
